# A real-world study of treatment patterns following disease progression in epithelial ovarian cancer patients undergoing poly-ADP-ribose polymerase inhibitor maintenance therapy

**DOI:** 10.1186/s13048-024-01381-9

**Published:** 2024-03-05

**Authors:** Nan Zhang, Hong Zheng, Yunong Gao, Tong Shu, Hongguo Wang, Yan Cai

**Affiliations:** Gynecology Department, Key Laboratory of Carcinogenesis and Translational Research, Peking University Cancer Hospital and Institute, Ministry of Education of People’s Republic of China, Beijing, 100142 China

**Keywords:** PARP, Epithelial ovarian cancer, BRCA, Maintenance therapy, Subsequent therapy

## Abstract

**Background:**

The efficacy of subsequent therapy after poly-ADP-ribose polymerase (PARP) inhibitor maintenance treatment has raised concerns. Retrospective studies show worse outcomes for platinum-based chemotherapy after progression of PARP inhibitor-maintenance therapy, especially in BRCA-mutant patients. We aimed to describe subsequent therapy in ovarian cancer patients after PARP inhibitor-maintenance therapy and evaluate their response to treatment. We focused on chemotherapy for patients with a progression-free interval (PFI) of ≥ 6 months after prior platinum treatment, based on BRCA status.

**Methods:**

We analyzed real-world data from Peking University Cancer Hospital, subsequent therapy after progression to PARP inhibitor-maintenance therapy for epithelial ovarian cancer between January 2016 and December 2022. Clinicopathological characteristics and treatment outcomes were extracted from medical records. The last follow-up was in May 2023.

**Results:**

A total of 102 patients were included, of which 29 (28.4%) had a germline BRCA1/2 mutation and 73 (71.6%) exhibited BRCA1/2 wild-type mutations. The PARP inhibitors used were Olaparib (*n* = 62, 60.8%), Niraparib (*n* = 35, 34.3%), and others (*n* = 5, 4.9%). The overall response rate (ORR) was 41.2%, and the median time to second progression (mTTSP) was 8.1 months (95%CI 5.8–10.2). Of 91 platinum-sensitive patients (PFI ≥ 6 months) after progression to PARP inhibitor-maintenance therapy, 65 patients subsequently received platinum regimens. Among them, 30 had received one line of chemotherapy before PARP inhibitor-maintenance therapy. Analysis of these 30 patients by BRCA status showed an ORR of 16.7% versus 33.3% and mTTSP of 7.1 (95% CI 4.9–9.1) versus 6.2 months (95% CI 3.7–8.3, *P* = 0.550), for BRCA-mutant and wild-type patients, respectively. For the remaining 35 patients who had received two or more lines of chemotherapy before PARP inhibitor-maintenance therapy, ORR was 57.1% versus 42.9%, and mTTSP was 18.0 (95% CI 5.0–31.0) versus 8.0 months (95% CI 4.9–11.1, *P* = 0.199), for BRCA-mutant and wild-type patients, respectively.

**Conclusion:**

No differences in survival outcomes were observed among patients with different BRCA statuses. Furthermore, for patients who had undergone two or more lines of chemotherapy before PARP inhibitor maintenance therapy, no negative effects of PARP inhibitors on subsequent treatment were found, regardless of BRCA status.

## Background

Poly-ADP-ribose polymerase (PARP) inhibitors (PARPi) have revolutionized the treatment of advanced epithelial ovarian, fallopian tube, and primary peritoneal adenocarcinoma. They work through the concept of “synthetic lethality” and have shown improved progression-free survival (PFS) and overall survival compared to chemotherapy alone [1–5]. Patients with BRCA1/2 mutation or homologous recombination deficiency (HRD) derive greater benefits from PARPi maintenance therapy. Thus, BRCA mutation and HRD status have become predictors of PARPi efficacy. However, concerns have arisen regarding the effectiveness of subsequent therapy after PARPi maintenance treatment.

The platinum-free interval (PFI), which indicates the time between the last platinum dose and relapse, is a crucial predictor of platinum sensitivity in relapsed patients [6]. The National Comprehensive Cancer Network guideline recommends platinum-based chemotherapy (PBC) for patients with a PFI of ≥ 6 months [7]. However, evidence suggests potential cross-resistance between PARPi and platinum, particularly in BRCA-mutated tumors [8]. Retrospective studies have reported lower objective response rates (ORR) in BRCA-mutated patients treated with Olaparib who had longer PFIs, indicating reduced efficacy [9]. Furthermore, an analysis of SOLO2 trial showed a significantly increased time to second progression (TTSP) with placebo compared to PBC in patients receiving Olaparib or placebo after progression [10]. In a Spanish multicentre real-world study of 111 patients who relapsed after PARPi maintenance therapy, those treated with PBC had an ORR of 41.9% and a median PFS of 6.6 months. In contrast, patients with BRCA mutations had a median PFS of only 3.5 months [11]. These findings challenge the traditional treatment paradigm of recurrent ovarian cancer after PARPi maintenance therapy based on PFI.

Therefore, we aimed to describe the subsequent therapy for patients with epithelial ovarian cancer who experienced disease progression after PARPi maintenance therapy. We also evaluated their response to subsequent treatment, focusing particularly on chemotherapy for patients with PFI ≥ 6 months, based on BRCA status.

## Materials & methods

### Study design and population

We retrospectively reviewed clinicopathological data from patients diagnosed and treated at Peking University Cancer Hospital between January 2016 and December 2022. The study included patients with histologically confirmed epithelial ovarian cancer, fallopian tube cancer, and primary peritoneal adenocarcinoma. Inclusion criteria were: (1) age ≥ 18 years; (2) confirmed diagnosis of epithelial ovarian cancer, fallopian tube cancer, or primary peritoneal adenocarcinoma; (3) received subsequent therapy after progression to PARPi maintenance therapy. We excluded patients who did not receive subsequent therapy or lacked follow-up data.

The Peking University Cancer Hospital & Institute Review Board approved this retrospective study.

### Data collection

Patient data including age at diagnosis, BRCA1/2 mutational status, histologic subtype, stage at diagnosis, the timing of surgery (primary or interval debulking surgery), surgical outcome, lines of chemotherapy before PARPi, type of PARPi used, duration of PARPi medication, PFI, details of subsequent therapy, the best response to subsequent treatment, progression data, and survival information, were collected from medical records. The last follow-up occurred in May 2023.

**Fig. 1:**
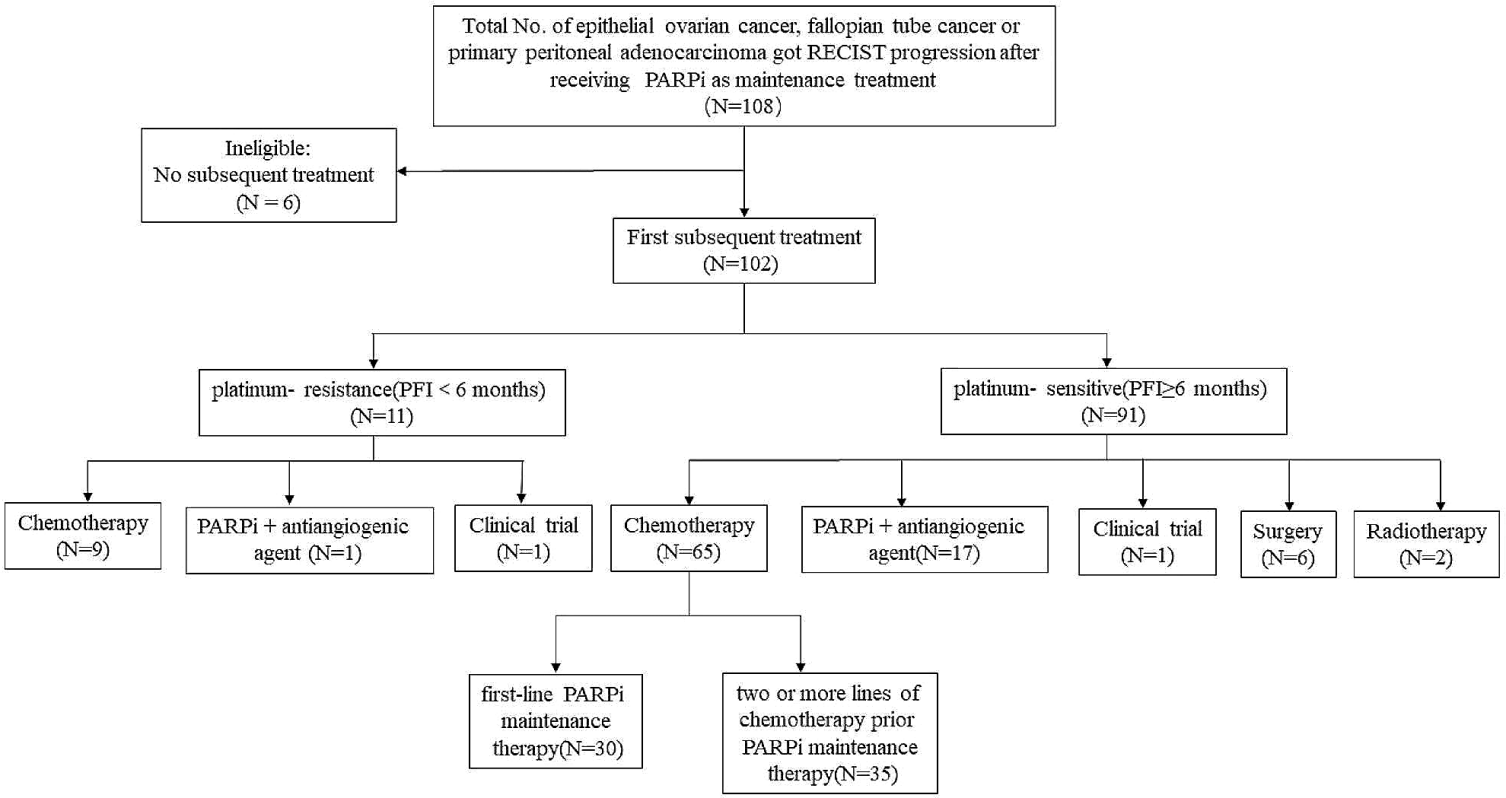
The inclusion/exclusion process and outline of the treatment patterns post PARPi maintenance therapy

To assess the effectiveness of chemotherapy following relapse in patients with a PFI of ≥ 6 months, we divided all patients into two subgroups: those with PFI < 6 months, referred to as the platinum-resistance group, and those with PFI ≥ 6 months, referred to as the platinum-sensitive (PS) group. Figure 1 shows the process of inclusion/exclusion and outlines the treatment patterns post-PARPi maintenance therapy.

### Outcomes

We defined the ORR as the proportion of patients achieving complete response (CR) or partial response (PR) and evaluated disease response according to Response Evaluation Criteria in Solid Tumors (RECIST1.1) or GCIG CA125 response criteria. TTSP was defined as the duration from initiating subsequent therapy post PARPi maintenance therapy to the second progression or death.

### Statistical analysis

Categorical variables were presented as percentages, while continuous variables were described as medians and ranges. Statistical significance was assessed using the χ^2^ test. TTSP was estimated using the Kaplan-Meier method, and differences between groups were evaluated using the log-rank test. The COX proportional hazards model was used to identify prognostic factors. Prognostic factors with *P* values < 0.1 in univariate analysis were further assessed in multivariate analysis. Statistical analysis was performed using SPSS 21.0 statistical package (SPSS Inc., Chicago, IL, USA).

## Results

### Basic clinical information

A total of 108 potentially eligible patients were screened, and ultimately, 102 patients were enrolled in the study. Six patients were excluded due to a lack of subsequent therapy. The detailed clinical characteristics of the included patients are presented in Table 1. The median duration of PARPi maintenance therapy in the cohort was 9.1 months, ranging from 2.0 to 43.6. At the last follow–up in May 2023, 84 patients had experienced disease progression after subsequent therapy, with a median TTSP (mTTSP) of 8.1 months (95%CI 5.8–10.2).

**Table 1:** The characteristics of all 102 included patients

Characteristics	No of patients (%)
Total patients	102
Median age (range) years	59 (26–80)
**BRCA1/2 status**	
BRCA WT	73 (71.6)
gBRCA1	22 (21.6)
gBRCA2	7 (6.9)
**Primary site**	
Ovary	91 (89.2)
Fallopian tube	8 (7.8)
Primary peritoneal	3 (2.9)
**Histological subtype**	
Serous	96 (94.1)
Mucinous	3 (2.9)
Clear cell	2 (2.0)
other	1 (1.0)
**Stage at diagnosis**	
I	4 (3.9)
II	5 (4.9)
III	69 (67.7)
IV	23 (22.5)
Unknown	1 (1.0)
**Timing of surgery**	
Primary	42 (41.2)
Interval	60 (58.8)
**Surgical outcome**	
R0	85 (83.3)
R1	16 (15.7)
Not optimal	1 (1.0)
**Lines of maintenance treatment before PARPi**	
1	46 (45.1)
2	39 (38.2)
≥ 3	17 (16.7)
**PARPi used**	
Olaparib	62 (60.8)
Niraparib	35 (34.3)
other	5 (4.9)

Among the patients, 52 (51%) received PBC, while 22 (21.6%) received non–platinum chemotherapy. Furthermore, 18 (17.6%) patients received a combination of PARPi and an antiangiogenic agent, two (2.0%) patients participated in a clinical trial, six (5.9%) patients underwent secondary debulking surgery, and two (2.0%) received radiotherapy. Regarding the best response to the subsequent therapy after PARPi maintenance therapy, the ORR was 41.2%, which six (5.9%) patients achieved CR, 36 (35.3%) achieved PR, 25 (24.5%) achieved stable disease (SD), and 35 (34.3%) achieved progressive disease (PD), as shown in Table 2.

**Table 2:** Subsequent treatment and response of 102 included patients

Treatment response	No of patients (%)
**Treatment post PARPi**	
Platinum-based chemotherapy	52 (51.0)
Non-platinum chemotherapy	22 (21.6)
PARPi + antiangiogenic agent	18 (17.6)
Clinical trial	2 (2.0)
Surgery	6 (5.9)
Radiotherapy	2 (2.0)
**Response to subsequent treatment of PARPi maintenance therapy**	
Complete response	6 (5.9)
Partial response	36 (35.3)
Stable disease	25 (24.5)
Progressive disease	35 (34.3)

Among 91 patients in the PS group, 65 received subsequent chemotherapy, 17 received PARPi + antiangiogenic agent treatment, one participated in a clinical trial, six underwent secondary debulking surgery, and two received radiotherapy. Among 65 patients who received subsequent chemotherapy in the PS group, based on the AE of previous chemotherapy and evaluation of the condition of each patient, clinicians adopted chemotherapy with a platinum-containing regimen for 52 patients and a non-platinum-containing regimen for 13patients.Of the 65 patients treated with chemotherapy, 30 received one line of chemotherapy before PARPi maintenance therapy that described as the “first-line PARPi maintenance therapy” group, while 35 received two or more lines that described as the “two or more lines of chemotherapy prior PARPi maintenance therapy” group, as shown in Fig. 1.

### Subgroup analysis for patients who received PARPi maintenance therapy after chemotherapy

**Fig. 2:**
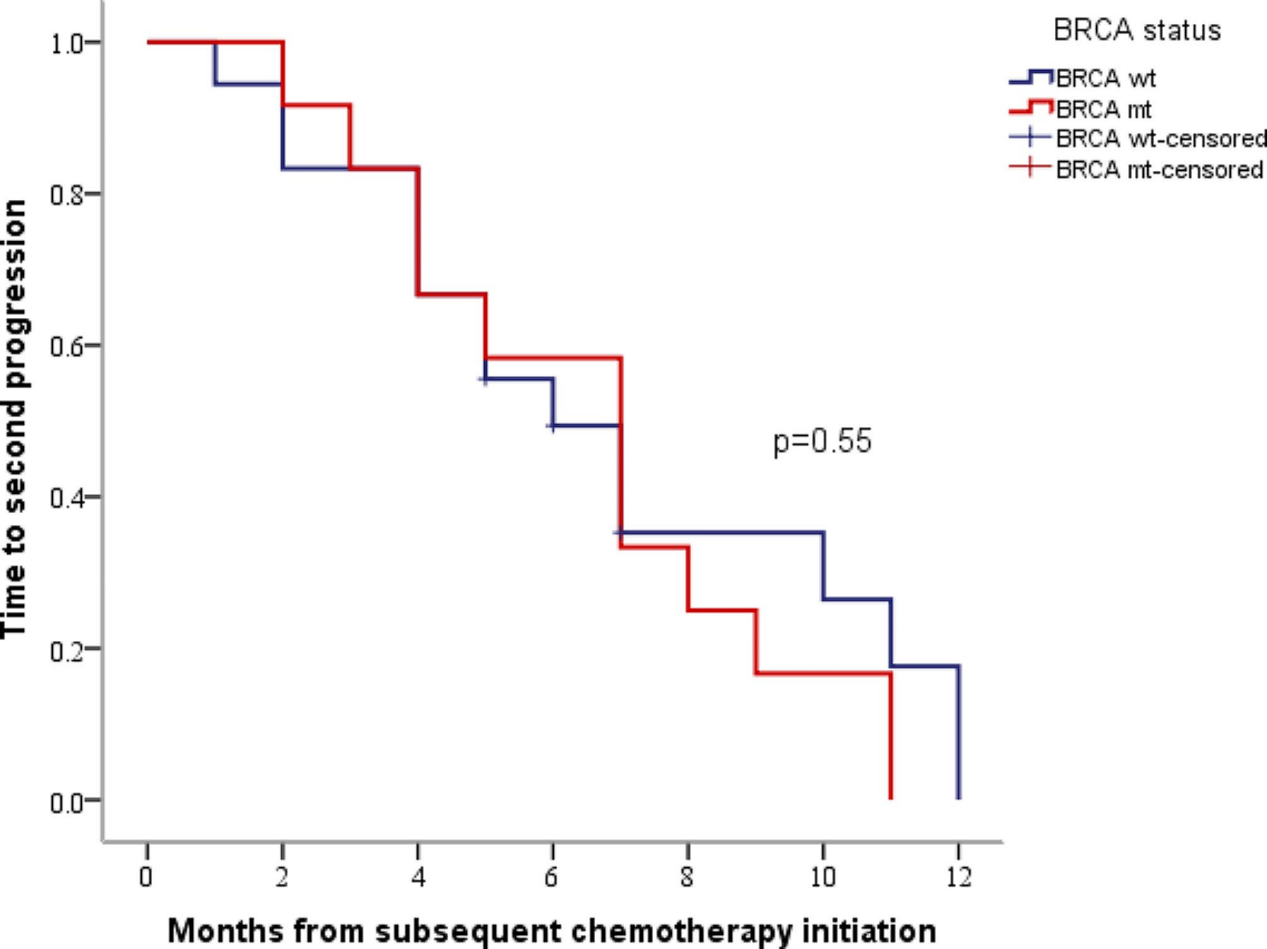
Kaplan-Meier estimates of TTSP in PS patients who received one line of subsequent chemotherapy before PARPi maintenance therapy based on BRCA status

An analysis of the subgroup of 30 patients who received one line of chemotherapy before PARPi maintenance therapy, stratified by BRCA status, revealed an ORR of 16.7% for BRCA mutant patients compared to 33.3% for BRCA wild–type patients. The mTTSP was 7.1 months (95%CI 4.9–9.1) for BRCA mutant patients and 6.2 months (95%CI 3.7–8.3) for BRCA wild–type patients, with no statistically significant difference (*P* = 0.550), as shown in Fig. 2. To examine the impact of PFI on efficacy, these patients were further divided into two subgroups: 6 ≤ PFI < 12 months and PFI ≥ 12 months. The outcomes of patients with 6 ≤ PFI < 12 months (*n* = 20) versus PFI ≥ 12 months (*n* = 10) were as follows: ORR was 15% versus 50%, and mTTSP was 5.1 months (95%CI 3.6–6.4) versus 10.0 months (95%CI 5.9–12.1), with a significant difference observed (*P* = 0.041). Patients with a PFI of 6 ≤ PFI < 12 months had inferior outcomes than those with PFI ≥ 12 months. Within the 6 ≤ PFI < 12 months group, the ORRs for BRCA mutant and BRCA wild–type patients were 0% versus 23.1%, with mTTSP of 4.2 months (95%CI 2.7–5.3) versus 5.1 months (95%CI 2.9–7.1) (*P* = 0.726). In the PFI ≥ 12 months, the ORR for BRCA mutant and BRCA wild–type patients were 40% versus 60%, with mTTSP of 9.1 months (95%CI 6.9–11.1) versus 10.1 months (95%CI 1.4–18.6) (*P* = 0.763). No significant differences in mTTSP were observed between different BRCA statuses within both subgroups (Table 3).

**Table 3:** The ORR and mTTSP of patients who received one line of chemotherapy before PARPi maintenance therapy based on BRCA status and PFI in the PS group

	PFI ≥ 6 months		6 ≤ PFI < 12 months		PFI ≥ 12 months	
	ORR (%)	mTTSP (95%CI) (month)	ORR (%)	mTTSP (95%CI) (month)	ORR (%)	mTTSP (95%CI) (month)
BRCA mutations (*n* = 12)	16.7	7.1 (4.9–9.1)	0	4.2 (2.7–5.3)	40	9.1 (6.9–11.1)
BRCA wild–type (*n* = 18)	33.3	6.2 (3.7–8.3)	23.1	5.1 (2.0–7.1)	60	10.1 (1.4–18.6)
Total (*n* = 30)	26.7	7.1 (5.4–8.6)	15	5.1 (3.6–6.4)	50	10.0 (5.9–12.1)

**Fig. 3:**
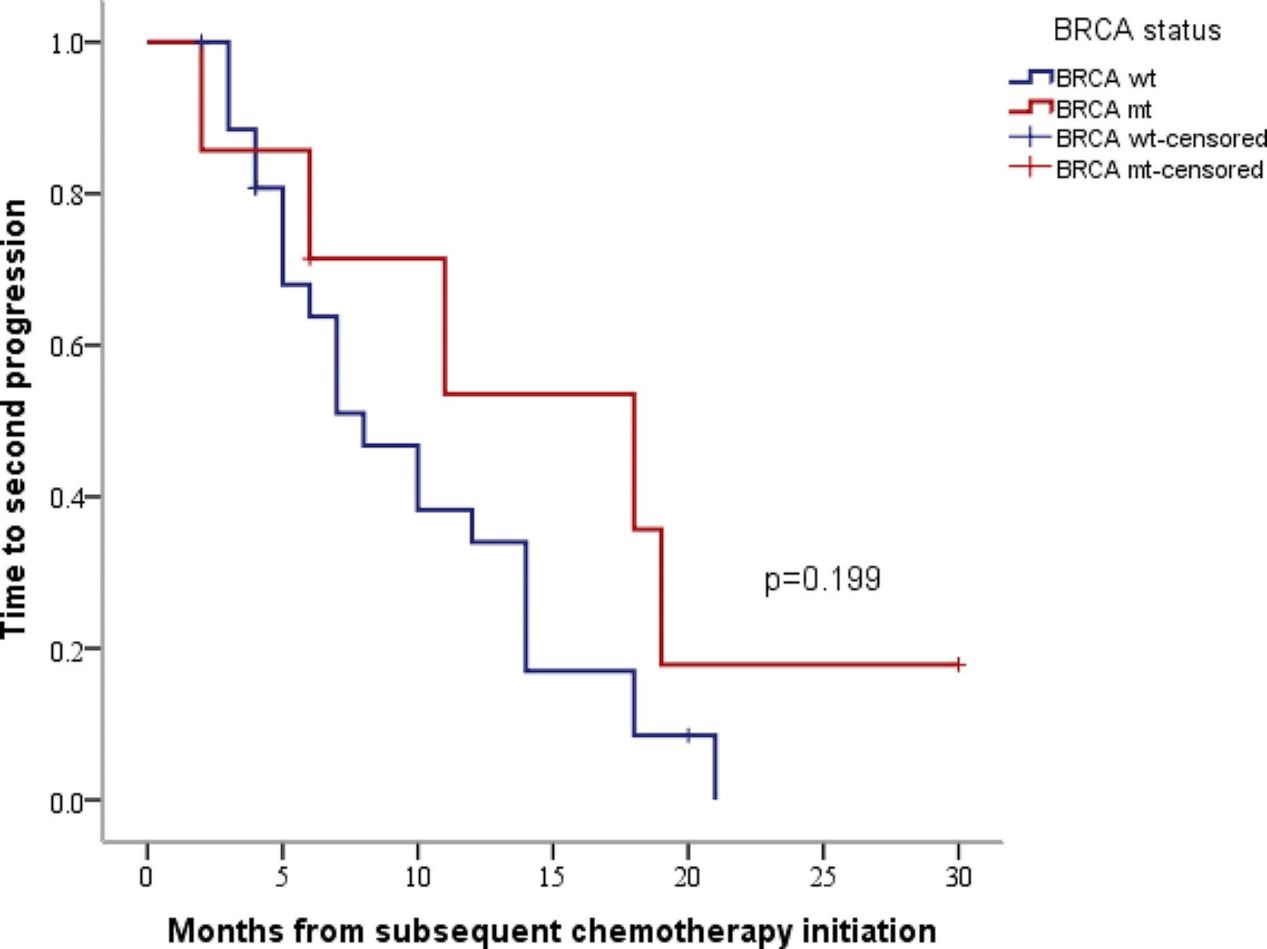
Kaplan-Meier estimates of TTSP in PS patients who received two or more lines of subsequent chemotherapy before PARPi maintenance therapy based on BRCA status

The analysis of the 35 patients who had received two or more lines of chemotherapy before PARPi maintenance therapy showed that ORR for BRCA mutant patients was 57.1% compared to 42.9% for BRCA wild–type patients, with mTTSP of 18.0 months (95%CI 5.0–31.0) versus 8.0 months (95%CI 4.9–1.1), respectively (*P* = 0.199) (Fig. 3). However, no significant difference in mTTSP was found between BRCA mutant and BRCA wild–type patients. The outcomes of patients with 6 ≤ PFI < 12 months (*n* = 13) versus PFI ≥ 12 months (*n* = 22) were as follows: ORR was 46.2% versus 45.5%, and mTTSP was 10.2 months (95%CI 5.6–14.4) versus 10.5 months (95%CI 4.8–15.2) (*P* = 0.684). In this group, no significant differences in mTTSP were found between patients with 6 ≤ PFI < 12 months and PFI ≥ 12 months. However, within the 6 ≤ PFI < 12 months group, the ORRs for BRCA mutant and BRCA wild-type patients were 50% versus 45.5%, with mTTSP of 19.0 months (95%CI 17.5–19.5) versus 7.4 months (95%CI 5.5–8.5) (*P* = 0.039). In the PFI ≥ 12 months group, the ORRs for BRCA mutant and BRCA wild–type patients were 60% versus 41.2%, with mTTSP of 10.5 months (95%CI 3.2–18.8) versus 10.5 months (95%CI 4.6–15.4) (*P* = 0.624) (Table 4).

**Table 4:** The ORR, mTTSP of patients who received two or more lines of chemotherapy before PARPi maintenance therapy based on BRCA status in the PS group

	PFI ≥ 6 months		6 ≤ PFI < 12 months		PFI ≥ 12 months	
	ORR (%)	mTTSP (95%CI) (month)	ORR (%)	mTTSP (95%CI) (month)	ORR (%)	mTTSP (95%CI) (month)
BRCA mutations (*n* = 7)	57.1	18.0 (5.0–31.0)	50	19.0 (17.5–19.5)	60	10.5 (3.2–18.8)
BRCA wild–type (*n* = 28)	42.9	8.0 (4.9–11.1)	45.5	7.4 (5.5–8.5)	41.2	10.5 (4.6–15.4)
Total (*n* = 35)	45.7	10.0 (4.9–15.2)	46.2	10.2 (5.6–14.4)	45.5	10.5 (4.8–15.2)

An analysis including BRCA status, duration of PARPi maintenance therapy (≥ 12 months or not), chemotherapy pattern (platinum–based or not), and chemotherapy combined with bevacizumab (yes or no) was performed for these 65 patients treated with chemotherapy in the PS group (Table 5). Duration of PARPi maintenance was found to be a significant factor for mTTSP in the univariate analysis (*P* = 0.029). However, this factor was not found to be independent in impacting mTTSP in the multivariate regression analysis (*P* = 0.059).

**Table 5:** Cox regression analysis of factors associated with mTTSP in 65 patients who received subsequent chemotherapy in the PS group

	Univariate analysis		Multivariate analysis	
	HR(95%CI)	P value	HR(95%CI)	P value
BRCA status (BRCA WT vs. BRCA mutation)	0.960(0.540–1.707)	0.889		
Chemotherapy combined with bevacizumab ( Yes vs. No)	0.766(0.444–1.321)	0.337		
chemotherapy pattern(platinum-based or not)	0.934(0.618–1.413)	0.748		
Duration of PARPi maintenance therapy(≥ 12months or not)	0.265(0.080–0.875)	0.029	0.572(0.320–1.021)	0.059

Among the 11 patients in the platinum-resistance group, nine received subsequent non–platinum chemotherapy, one received PARPi + antiangiogenic agent, and one received treatment as part of a clinical trial. Four patients were BRCA mutant, and seven were BRCA wild–type. The ORR was 54.5%, and mTTSP was 5.0 months (95%CI 3.0–7.0).

## Discussion

Clinical trials have demonstrated that PARPi maintenance therapy can significantly prolong PFS in patients with both primary and recurrent ovarian cancer [1–5]. PARPi slows the need for subsequent treatment and extends the PFI, particularly in patients with BRCA mutants. However, like other therapies, resistance to PARPi eventually develops. The exact mechanism behind PARPi resistance remains unclear, but potential factors include reversion mutations, methylation events, and restoration of HRD through combinations and targeting replication stress [12].

Notably, PARPi and platinum agents may share some resistance mechanisms, explaining the observed cross-resistance between these two drugs [13]. Our retrospective study focused on the subsequent therapy administered to patients with epithelial ovarian cancer who experienced disease progression after PARPi maintenance therapy. Furthermore, adhering to current treatment guidelines for platinum-sensitive recurrent ovarian cancer patients and seeking to investigate the presence of cross-resistance, we conducted a detailed analysis of re-chemotherapy in patients with a PFI of at least 6 months. Among this subgroup of 65 patients, most were treated with a platinum-containing regimen (52 out of 65, accounting for 80%). Moreover, these patients were further categorized based on the number of previous chemotherapy lines they had received.

Several retrospective studies have reported on the subsequent chemotherapy in patients who received two or more lines of chemotherapy before PARPi maintenance therapy. Most of these studies [9–11] indicated a potential negative impact of PARPi on the efficacy of subsequent platinum-based chemotherapy, particularly in the BRCA-mutant population. However, these studies did not include patients who experienced relapse after first-line maintenance therapy with PARPi, and limited data are available for patients with wild-type BRCA. Therefore, we conducted this real-world study to include patients who relapsed after first-line maintenance therapy with PARPi and those who relapsed after second-line and above maintenance therapy to explore the effects of PARPi on different patient populations.

Of the 30 patients who received first-line PARPi maintenance therapy before subsequent chemotherapy, the ORR was 26.7%, and mTTSP was 7.1 months. These results were lower than what has historically been reported for similar populations. For instance, the ICON4 study, a multicenter randomized controlled trial of platinum-containing chemotherapy in platinum-sensitive relapsed ovarian cancer patients, in which more than 90% of enrolled patients experienced first relapse, reported ORR of 54% and 66%, with a PFS of 10 months and 13 months, respectively [14]. Similarly, the GOG213 study, another phase 3 multicenter randomized controlled trial in platinum-sensitive relapsed ovarian cancer, showed an ORR of 59% and a PFS of 10.4 months in the platinum-containing chemotherapy group [15]. Notably, the proportion of patients with a PFI of at least 12 months in our study was only 33.3%, compared to 73–77% in ICON4 and 69% in GOG213, which may have contributed to the poorer survival outcomes observed in our study.

Furthermore, ORR was unexpectedly poor in the 6 ≤ PFI < 12 months subgroup, especially in BRCA mutant patients. The MITO-8 [16] was an international, multicenter, randomized, open-label phase III trial in which patients with partially platinum-sensitive ovarian cancer were treated with either PBC or non-PBC (NPBC). The ORR was 56% in the PBC group and 43% in the NPBC group, regardless of BRCA status. The median PFS was 9.0 months (95% CI, 7.6 to 10.4 months) in the PBC group and 5.0 months (95% CI, 4.1 to 5.9 months) in the NPBC group. In the subgroup of patients with a PFI between 6 and 12 months, we found a lower ORR of 15%. The PFS (5.1 months, 95% CI,3.6 to 6.4 months) in our study was lower than the PBC group in the MITO-8 study. Given that most patients in our subgroup received PBC treatment, our survival outcomes for this subgroup of patients were not satisfactory. Disease progression occurred in these patients after a short period of PARPi maintenance therapy, indicating resistance to PARPi, and insensitivity to subsequent chemotherapy may suggests the possibility of cross-resistance between PARPi and platinum agents to some extent.

Although we observed a lower-than-expected ORR in the BRCA mutant patients, there was no statistically significant difference in mTTSP between the BRCA mutant and wild-type groups. In the subgroup with a PFI of at least 12 months, the ORR (50%) and mTTSP (10.0 months) were comparable to historical outcomes for similar patient populations. We also did not observe any differences in survival outcomes among patients with different BRCA status in this subgroup. It is worth noting that all the patients in our study had experienced recurrence during first-line maintenance treatment. In contrast, in the real world, many patients are still undergoing first-line maintenance treatment. This may have introduced selection bias into our study. Further research is needed to determine whether PARPi negatively impacts the efficacy of subsequent chemotherapy post-first-line maintenance treatment and whether this effect is consistent across different BRCA states. In the PS group, which consisted of 35 patients who had received two or more lines of chemotherapy before PARPi maintenance therapy, the ORR was 45.7%, comparable to the results of a Spanish real-world study (41.9%) [11]. However, the mTTSP was better in our study (10.0 months vs.6.6 months). Stratifying by PFI, we found that 62.9% of patients in our subgroup with PFI > 12 months had better outcomes than the 40.7% in the Spanish real-world study. Moreover, we didn’t observe worse outcomes or significant differences between BRCA mutant and BRCA wild-type patients. It’s worth noting that only 20% of patients in our subgroup had BRCA mutations. In contrast, the Spanish real-world study had a higher proportion (44.4%), which may explain why we failed to detect survival outcome differences based on BRCA status. In the SOLO2 post-hoc analysis, the mTTSP in the Olaparib group was 7.0 months [10], but all the patients included in this study had BRCA mutations. Our study included a higher proportion of BRCA wild-type patients and could not find a negative effect of PARPi maintenance therapy on subsequent chemotherapy. However, it is important to acknowledge that the small number of cases with BRCA mutations in the 6 ≤ PFI < 12 months group could have introduced significant bias into our findings. In addition, due to the selection bias, this subgroup may be a special population that many times was susceptible to platinum.

## Conclusion

The outcomes for patients who received first-line PARPi maintenance therapy before subsequent chemotherapy were inferior to historically reported results for similar patients who did not receive PARPi maintenance therapy previously. Nevertheless, we observed no differences in survival outcomes based on BRCA status. In patients who had received two or more lines of chemotherapy before PARPi maintenance therapy, we found no detrimental effects of PARPi on subsequent treatment, regardless of BRCA status.

However, as a single-center retrospective study, we could not draw definitive conclusions from the limited number of cases regarding the development of platinum cross-resistance in patients receiving PARPi as maintenance therapy and whether BRCA status influenced treatment efficacy. We hypothesized that the mechanism of PARPi resistance may vary among different populations. The PFI remains a crucial factor in guiding treatment choices for relapsed patients. The impact of PARPi on subsequent chemotherapy is still unclear and necessitates further investigation.

## References

[1] Moore K, Colombo N, Scambia G, Kim BG, Oaknin A, Friedlander M, Engl (2018). Maintenance Olaparib in patients with newly diagnosed Advanced Ovarian Cancer. N. J Med.

[2] Pujade-Lauraine E, Ledermann JA, Selle F, Gebski V, Penson RT, Oza AM (2017). Olaparib tablets as maintenance therapy in patients with platinum-sensitive, relapsed ovarian cancer and a BRCA1/2 mutation (SOLO2/ENGOT-Ov21): a double-blind, randomised, placebo-controlled, phase 3 trial. Lancet Oncol.

[3] Mirza MR, Monk BJ, Herrstedt J, Oza AM, Mahner S, Redondo A, Engl (2016). Niraparib Maintenance Therapy in Platinum-Sensitive, recurrent ovarian Cancer. N. J Med.

[4] González-Martín A, Pothuri B, Vergote I, DePont Christensen R, Graybill W, Mirza MR, Engl (2019). Niraparib in patients with newly diagnosed Advanced Ovarian Cancer. N. J Med.

[5] Coleman RL, Oza AM, Lorusso D, Aghajanian C, Oaknin A, Dean A (2017). Rucaparib maintenance treatment for recurrent ovarian carcinoma after response to platinum therapy (ARIEL3): a randomised, double-blind, placebo-controlled, phase 3 trial. Lancet.

[6] Fung-Kee-Fung M, Oliver T, Elit L, Oza A, Hirte HW, Bryson P (2007). Optimal chemotherapy treatment for women with recurrent ovarian cancer. Curr Oncol.

[7] National Comprehensive Cancer Network. Ovarian Cancer including fallopian Tube Cancer and primary peritoneal Cancer. (Version 1.2023). http://www.nccn.org/professionals/physician_gls/pdf/Ovarian.pdf.

[8] Bartoletti M, Cecere SC, Musacchio L, Sorio R, Puglisi F, Pignata S (2021). Recurrent ovarian cancer in the era of poly-ADP ribose polymerase inhibitors: time to re-assess established clinical practices. ESMO Open.

[9] Cecere SC, Giannone G, Salutari V, Arenare L, Lorusso D, Ronzino G (2020). Olaparib as maintenance therapy in patients with BRCA 1–2 mutated recurrent platinum sensitive ovarian cancer: real world data and post progression outcome. Gynecol Oncol.

[10] Frenel JS, Kim JW, Aryal N, Asher R, Berton D, Vidal L (2022). Efficacy of subsequent chemotherapy for patients with BRCA1/2-mutated recurrent epithelial ovarian cancer progressing on olaparib versus placebo maintenance: post-hoc analyses of the SOLO2/ENGOT Ov-21 trial. Ann Oncol.

[11] Romeo M, Gil-Martín M, Gaba L, Teruel I, Taus Á, Fina C et al. Multicenter Real-World Data of subsequent chemotherapy after progression to PARP inhibitors in a maintenance relapse setting. Cancers (Basel). 2022;14(18):4414. 10.3390/cancers14184414. 10.3390/cancers14184414 PMC9497128 36139574

[12] Washington CR, Moore KN (2022). Resistance to poly (ADP-Ribose) polymerase inhibitors (PARPi): mechanisms and potential to reverse. Curr Oncol Rep.

[13] Kyo S, Kanno K, Takakura M, Yamashita H, Ishikawa M, Ishibashi T et al. Clinical Landscape of PARP inhibitors in Ovarian Cancer: Molecular mechanisms and clues to overcome resistance. Cancers (Basel). 2022;14(10):2504. 10.3390/cancers14102504. 10.3390/cancers14102504 PMC9139943 35626108

[14] Parmar MK, Ledermann JA, Colombo N, du Bois A, Delaloye JF, Kristensen GB (2003). Paclitaxel plus platinum-based chemotherapy versus conventional platinum-based chemotherapy in women with relapsed ovarian cancer: the ICON4/AGO-OVAR-2.2 trial. Lancet.

[15] Coleman RL, Brady MF, Herzog TJ, Sabbatini P, Armstrong DK, Walker JL (2017). Bevacizumab and paclitaxel-carboplatin chemotherapy and secondary cytoreduction in recurrent, platinum-sensitive ovarian cancer (NRG Oncology/Gynecologic Oncology Group study GOG-0213): a multicentre, open-label, randomised, phase 3 trial. Lancet Oncol.

[16] Pignata S, Scambia G, Bologna A, Signoriello S, Vergote IB, Wagner U (2017). Randomized Controlled Trial Testing the efficacy of platinum-free interval prolongation in Advanced Ovarian Cancer: the MITO-8, MaNGO, BGOG-Ov1, AGO-Ovar2.16, ENGOT-Ov1, GCIG Study. J Clin Oncol.

